# Investigation on the Surface Integrity of 40Cr Steel Machined by Rotary Ultrasonic Flank Milling

**DOI:** 10.3390/mi15020189

**Published:** 2024-01-26

**Authors:** Shuaijun Zhu, Yijia Sun, Feng Wang, Hu Gong

**Affiliations:** 1 State Key Laboratory of Precision Measurement Technology and Instruments, School of Precision Instrument and Optoelectronic Engineering, Tianjin University, Tianjin 300222, China; zhushuaij@tju.edu.cn (S.Z.); wangfeng1995@tju.edu.cn (F.W.); 2 Tianjin Key Laboratory for Control Theory & Applications in Complicated Industry Systems, Tianjin University of Technology, Tianjin 300382, China

**Keywords:** rotary ultrasonic machining, milling, surface quality, cutting mechanism

## Abstract

Rotary Ultrasonic Machining (RUM) stands as a crucial method for machining hard and brittle materials. However, for machining hard-to-machine metal, it continues to face many challenges due to the complex vibration of the milling tool. Flank milling is an efficient method for machining complex parts, such as blisks and impellers, which have been widely used in aerospace field. However, current research is more focused on rotary ultrasonic end milling. In this context, we will study the surface integrity of rotary ultrasonic flank milling 40Cr steel using a self-developed RUM system. We delve into exploring the impacts of tool vibration on surface morphology, residual stress, and micro-hardness of the workpiece under various process parameters. The experimental findings reveal that rotary ultrasonic flank milling, in contrast to traditional flank milling techniques, significantly diminishes the surface roughness by about 40%. The reasons for the reduction of surface roughness are analyzed from the point of view of the cutting force. The surface roughness appears to be notably linked to both the average cutting force and the frequency domain characteristics. In addition, the experimental results indicate that rotary ultrasonic flank milling demonstrates the capacity to elevate the micro-hardness of the machined surface.

## 1. Introduction

40Cr steel, characterized by high strength, high hardness, excellent wear resistance, corrosion resistance, fatigue performance, and a certain degree of toughness, is widely used in the aerospace and aviation fields. It is also utilized in the manufacturing of critical components such as rolling elements and bearing sleeves. There are some problems in the machining process, such as a large cutting force, a high cutting temperature, and serious tool wear, which makes the machining quality control difficulty and manufacturing cost of parts significantly increase. To address these challenges to some extent, researchers have explored various new machining processes, among which RUM stands out as a key method [1,2,3]. RUM is a non-traditional machining technique that integrates ultrasonic vibration with conventional milling processes. In machining ductile metal materials, cutting tools featuring spiral cutting edges—such as milling cutters and twist drills—are commonly employed [4]. For the tool with a helical cutting edge, the axial ultrasonic conduction will cause complex spatial vibration of the cutting edge due to the influence of the helical structure [5]. The increasing diversity in processing demands has led to a wider variety of types and structures for metal cutting tools. The resonant frequency, amplitude, phase, and vibration direction of the tool are intricately linked to the spiral structure of its cutting edge. Hence, compared with a simple structure tool, it is more difficult to study the mechanism of rotary ultrasonic machining in ductile metal materials with this kind of complex structure tool. In certain published documents, the superposition of ultrasonic vibration has been observed to positively impact the processing of metal materials, as evidenced by reduced cutting forces, diminished tool wear, and enhanced surface quality [6]. However, contradictory findings have also been reported, suggesting an adverse effect [7]. At present, it is difficult to give a convincing and reasonable explanation for these phenomena in theory. Deep and systematic theoretical investigations into the complex tool vibration’s mechanism and its impact on processing outcomes are essential for the effective design and control of rotary ultrasonic systems geared towards enhancing metal material machining.

The study of various influencing factors on process characteristics primarily encompasses key parameters such as cutting force, machining temperature, surface quality, and tool wear. The process characteristics in RUM are influenced by several factors, such as material properties, cooling conditions, tool parameters, and process settings. Surface quality serves as a paramount metric for assessing machining quality, often referred to as surface integrity. This integrity comprises two primary facets [8]: the first pertains to the geometric form of the workpiece’s outermost surface, while the second involves the mechanical, physical, and chemical properties of the surface layer at a specific depth. Given the multifaceted nature of surface quality, academic research on the surface characteristics of metal workpieces in rotary ultrasonic machining is extensive. This research predominantly centers on surface morphology, microhardness, and residual stress.

According to the research on process parameters of RUM, Verma et al. [9] found in rotary ultrasonic milling that during the contact between the tool and workpiece, the dynamic cutting force suddenly rises due to the impact between the tool and workpiece. With the increase in ultrasonic power, the intensity of this impact also increases, further leading to higher instantaneous cutting forces. Lian et al. [10] discovered that selecting an appropriate ultrasonic vibration amplitude in rotary ultrasonic machining results in smaller surface roughness compared to conventional micro-milling. They analyzed the impact of different ultrasonic amplitudes on surface roughness. The results indicate an optimal parameter for surface roughness concerning the amplitude of ultrasonic vibration. Marcel et al. [11] studied the surface quality parameters of ultrasonic-assisted milling of EN AW 5083 non-hardenable aluminum alloy. They achieved the best machining surface parameters at a spindle speed of 6000 rpm. Additionally, the roughness was almost independent of the feed rate. Yin et al. [12] studied the effect of vibration on the surface integrity of Inconel 718 and compared it with conventional milling. The results indicated that, compared to conventional milling processes, the ultrasonic peening milling technique resulted in better surface quality, fewer surface defects, a thicker stress-affected layer, and an enhanced machined surface, leading to a 16.1-fold increase in fatigue life. Gao et al. [13] investigated the influence of ultrasonic amplitude on three-component cutting forces, cutting temperature, surface morphology, and three-dimensional surface roughness. The results indicate that the cutting forces in the longitudinal and feed directions decrease with increasing amplitude, while the average and maximum cutting temperatures increase with larger amplitudes. When the ultrasonic amplitude reaches 6 µm, the surface quality is optimal. Zhang et al. [14] demonstrated that through rotary ultrasonic milling, a regular, extensive micro-vibration texture existed on the surface, characterized by separated ridge-like structures. As the cutting speed and vibration amplitude increased, the surface roughness also increased. At lower cutting speeds, both the subsurface deformation layer thickness and microhardness values increased. However, when the cutting speed reached 160 m/min, the hardening effect gradually diminished. At lower cutting speeds, surface residual compressive stress decreased gradually with the vibration amplitude, whereas at higher cutting speeds, the opposite was observed. Xie et al. [15] found that in rotary ultrasonic milling, an increased feed rate typically correlates with rising surface roughness, while the effect of ultrasonic amplitude on surface roughness exhibits no consistent trend. To effectively minimize surface roughness, optimal synchronization between rotation speed and ultrasonic amplitude is crucial. Kadivar et al. [16] observed that in rotary ultrasonic drilling, enhancing the spindle speed, decreasing the feed rate, and appropriately increasing tool amplitude effectively reduce hole wall surface roughness. To fully leverage the benefits of rotary ultrasonic drilling, aligning vibration, cutting, and tool parameters according to distinct processing methods becomes imperative. Optimizing the cutting edge’s motion trajectory significantly enhances workpiece surface quality. Regarding the impact of rotary ultrasonic machining on workpiece surface residual stress and microhardness, Ying et al. [17] demonstrated that the superposition of ultrasonic vibration enhances the residual compressive stress and improves surface microhardness. Particularly at lower cutting speeds, increased tool vibration correlates with escalated residual compressive stress and surface microhardness, contributing to enhanced fatigue strength and wear resistance of the workpiece. Barani et al. [18], in their study on the influence of ultrasonic vibration on surface roughness, observed that, compared to traditional drilling, rotary ultrasonic drilling typically enhances chip breaking and removal during the machining process. This method reduces scratching caused by chips on the hole wall and minimizes the formation of built-up edges. The superposition of the tool vibration helps to reduce the surface roughness of the drilled hole. Ultrasonic vibration, when applied in milling, exhibits varied effects on surface roughness across different experiments. Compared with conventional milling, the rotary ultrasonic milling method may increase the surface roughness, but it may also decrease the surface roughness. The inconsistency of research conclusions may be related to the matching of material and workpiece parameters. Upon studying the machined surface morphology, scholars have observed contrasting effects: On one hand, the addition of ultrasonic vibration effectively diminishes surface defects such as feed tool marks and residual burrs, contributing to reduced surface roughness. Conversely, ultrasonic vibration may introduce micro-texturing on the machined surface, leading to an increasing trend in surface roughness. It’s essential to note that surface roughness is influenced by multiple factors.

This paper will employ a typical helical end milling cutter in conducting rotary ultrasonic flank milling experiments on metal materials using a self-developed rotary ultrasonic machining system. The study explores the influence of tool vibration states on surface morphology, residual stress, and microhardness of the workpiece under various process parameters. These findings offer valuable insights into applying the rotary ultrasonic machining method to milling metal structural parts.

## 2. Experiment

### 2.1. Experimental Setup

**Figure 1:**
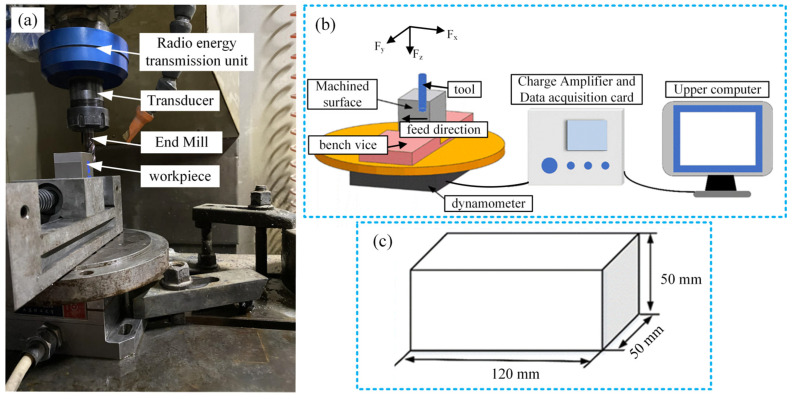
(**a**) Experimental device for rotary ultrasonic flank milling; (**b**) Cutting force measurement system; (**c**) Schematic diagram of workpiece.

**Figure 2:**
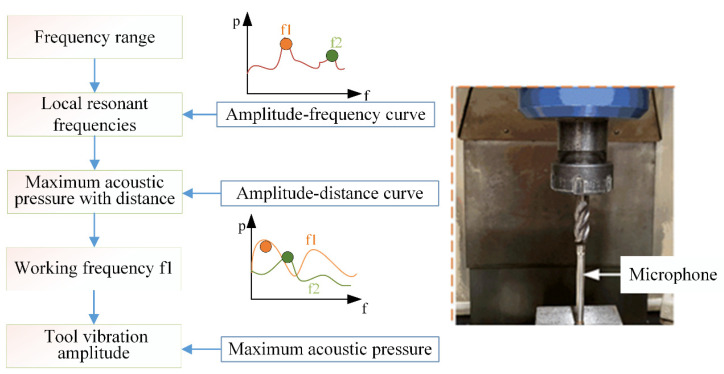
Acoustic characterization of the tool for ultrasonic vibration.

Figure 1a illustrates the experimental setup for rotary ultrasonic end milling. The experiment employs the flank milling method to process the workpiece, primarily utilizing the circumferential surface’s side edge of the milling cutter for machining. During machining, the tool axis aligns parallel to the machined surface, mitigating the influence of axial vibration on the workpiece surface. The tool parameters are listed in Table 1, and the FLY 2800SP cutting fluid is used for lubrication and cooling. The experimental machine tool is the XH714D vertical machining center, equipped with a self-developed rotary ultrasonic vibration system. The experiment employs a workpiece made of 40Cr modulated steel, featuring a rectangular structure measuring 50 mm in length, 50 mm in width, and 120 mm in height, as depicted in Figure 1c. Milling involves using the climb milling method to remove material from the workpiece. In climb milling, the cutting thickness of each milling cutter gradually increases from small to large with each cutting edge. The force exerted in the direction of the spindle is relatively minimal, reducing the wear on the machine and tool while minimizing damage to the workpiece surface. This method can improve the surface roughness of the machining surface, as shown in Figure 2.

Prior to machining, it is necessary to calibrate the ultrasonic vibration state of the tool. Considering the complex structure of the bottom edge of the milling tool, it is hard or even inoperable for the optical methods to be used to measure the displacement of the non-continuous surface because the surface with a complex profile could potentially shade the laser spot. Hence, the tool’s vibration amplitude was characterized by the acoustic pressure radiated by the tool’s ultrasonic vibration. The details of the acoustic characterization method for tool ultrasonic vibration can be referred to in our previous literature [19]. The working frequency of the milling tool was determined, and the variations in tool vibration amplitude were described quantitatively by measuring the radiated acoustic pressure. Figure 2 shows the setup for the tool for ultrasonic vibration characterization. A free-field condenser microphone (MNP41, SKC Acoustics Technology Corporation, Beijing, China) was set under the bottom of the milling tool along the tool axis to measure radiated acoustic pressure by the drill bit. Firstly, the working frequency of the tool is determined as 39.2 kHz by measuring the maximum sound pressure amplitude radiating along the tool axis. Subsequently, the magnitude of tool vibration is adjusted by modulating the power supply voltage, quantified by the maximum sound pressure amplitude radiating along the tool axis. In traditional milling processes where the tool does not undergo ultrasonic vibration, the radiated sound pressure amplitude measures 0 Pa.

### 2.2. Experimental Design

**Figure 3:**
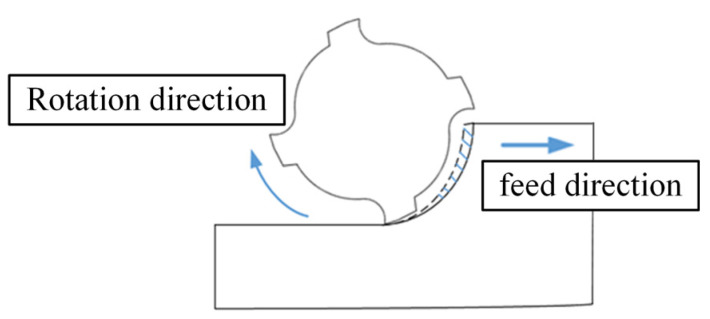
Schematic diagram of down milling.

A three-way piezoelectric dynamometer (YDC-III09, Dalian University of Technology, Dalian, China), charge amplifier, and data acquisition card were used to collect cutting force signals in real time during the experiment, as shown in Figure 1b. In the figure, the *X*-axis of the dynamometer represents the feed direction of the end milling machine, while the *Y*-axis denotes the cutting direction of the end milling machine. The *Z*-axis corresponds to the axial direction of the tool. Milling is performed using the climb milling method to remove material from the workpiece. During climb milling, the cutting thickness of each milling cutter gradually increases from small to large. The force exerted in the direction of the spindle is relatively minimal, reducing wear on the machine and tool. Simultaneously, this method causes minimal damage to the workpiece surface, which helps improve the surface roughness of the machined surface, as visually depicted in Figure 3. Prior to machining, calibrating the ultrasonic vibration state of the tool is essential. Initially, the working frequency of the tool, set at 39.2 kHz, is established by measuring the maximum sound pressure amplitude emitted along the tool’s axis. Subsequently, adjusting the power supply voltage allows for alterations in the magnitude of the tool’s vibration, enabling a quantitative characterization of the maximum sound pressure amplitude emitted along the tool’s axis. For traditional milling processes, the tools do not have ultrasonic vibration, and the radiated sound pressure amplitude is 0 Pa. In order to systematically explore the impact of tool vibration state on the surface quality of the workpiece machined by milling, a full factorial experimental design method was used to perform rotary ultrasonic milling experiments under different conditions of feed per tooth, axial cutting depth, and sound pressure amplitude. Table 2 shows the detailed process parameters for traditional flank milling and rotary ultrasonic milling experiments. All milling processes were carried out with the existence of cutting fluid.

## 3. Analysis of Results

### 3.1. Comparison of Surface Topography Machined by Conventional Flank Milling and Rotary Ultrasonic Flank Milling

**Figure 4:**
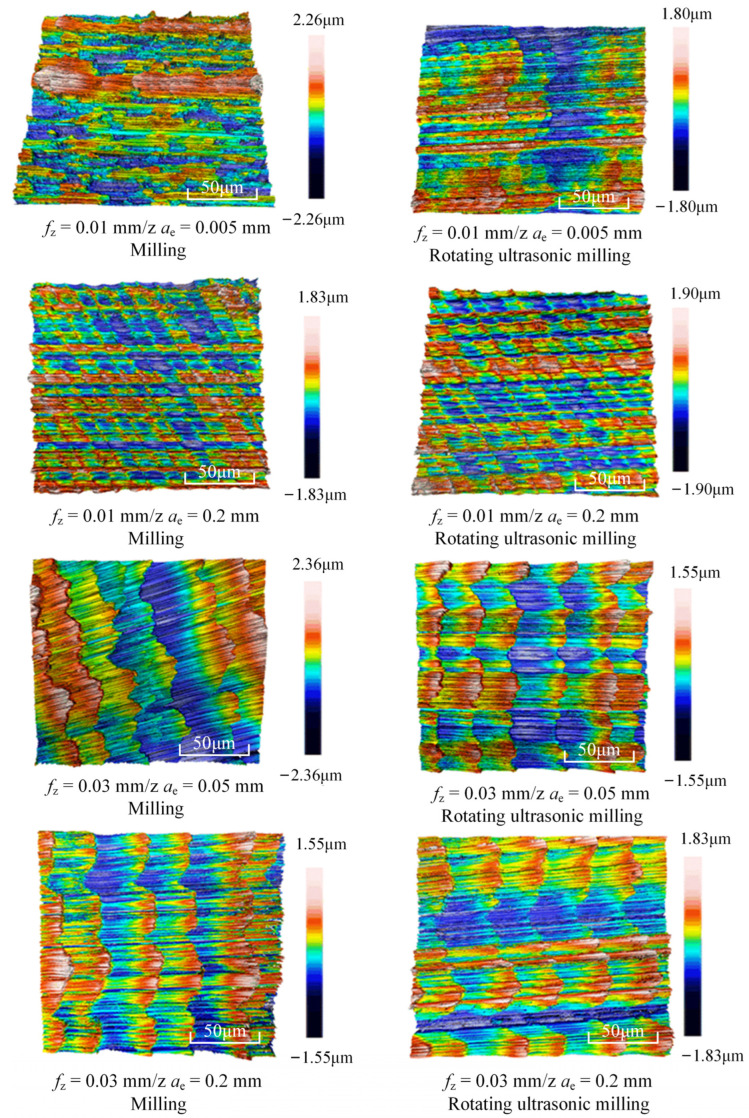
Three-dimensional topography of the machined surface by conventional flank milling and rotary ultrasonic flank milling.

Table 3 and Figure 4 show the surface topography of the workpiece obtained by conventional flank milling and rotary ultrasonic flank milling. It can be seen from the figure that there are obvious tool marks on the surface of the workpiece obtained by traditional flank milling, as well as defects such as furrows and scales with different depths and widths. In the rotary ultrasonic milling process, the repeated ironing and extrusion of the vibrating side edge on the workpiece surface make the surface texture distribution more uniform and regular, and the surface defects obtained by the rotary ultrasonic milling process are significantly reduced.

Upon comparing the surface topographies depicted in Figure 3 and Figure 4 for varying feed rates and cutting depths, it’s evident that during flank milling with a feed rate per tooth of 0.01 mm/z and a radial cutting depth of 0.05 mm, the regularly distributed tool marks resulting from the tool’s feed motion are less pronounced. However, the surface topography appears relatively rougher under these conditions. This is mainly due to the fact that, at a relatively small depth of cut, the workpiece material is primarily removed through extrusion deformation. In this scenario, the mutual compression between the cutting edge and the workpiece surface makes the cutting edge surface more susceptible to built-up edges. As the built-up edges attached to the cutting edge surface grow and detach, they often carve longitudinal grooves of varying depths and widths on the machined surface. Moreover, cold welding on the machined surface may result in burr formation, thereby leading to relatively poor surface uniformity. With a radial cutting depth increased to 0.2 mm, the surface topography’s uniformity decreases. In comparison to conventional flank milling with identical parameters, the vibration-induced ironing effect on the workpiece surface reduces the residual height on adjacent machined surfaces. Consequently, the workpiece processed with ultrasonic vibration appears relatively more uniform.

**Figure 5:**
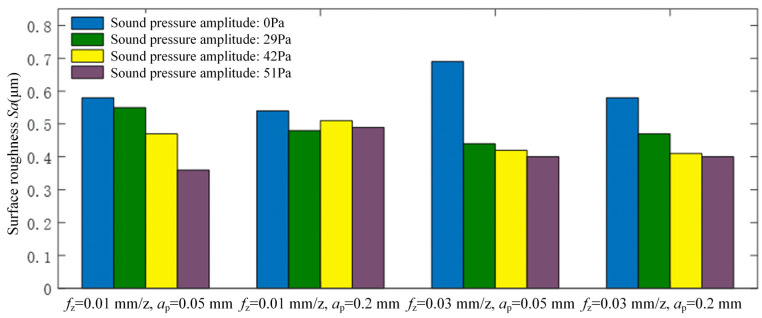
Influence of surface roughness *Sa* on the machined surface by conventional flank milling and rotary ultrasonic flank milling.

Figure 5 illustrates the comparison results of surface roughness (*Sa*) between machined surfaces produced by traditional flank milling and rotary ultrasonic flank milling. It can be seen from the figure that the rotary ultrasonic flank milling method significantly reduces workpiece surface roughness compared to traditional flank milling, showcasing a reduction of approximately 40%. Within rotary ultrasonic flank milling, the surface roughness of the workpiece tends to decrease with higher amplitudes of ultrasonic vibration. Hence, it can be inferred that greater ultrasonic vibration amplitude is advantageous for reducing workpiece surface roughness in flank milling operations.

### 3.2. Effect of Cutting Force on Surface Roughness of Workpiece in Rotary Ultrasonic Milling Process

Flank milling involves substantial material removal, consequently resulting in comparatively higher cutting forces. Given the direct correlation between cutting forces and surface topography formation, exploring the relationship between cutting force and surface roughness is a fundamental aspect. This study serves as essential groundwork for understanding the mechanism and devising strategies to enhance surface roughness. The following work will focus on the analysis of the influence of cutting force on the surface roughness of the workpiece in rotary ultrasonic flank milling.

**Figure 6:**
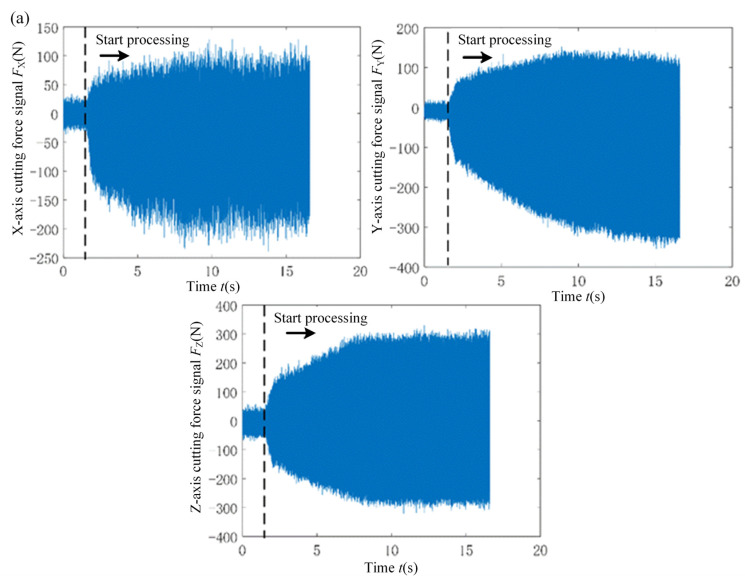

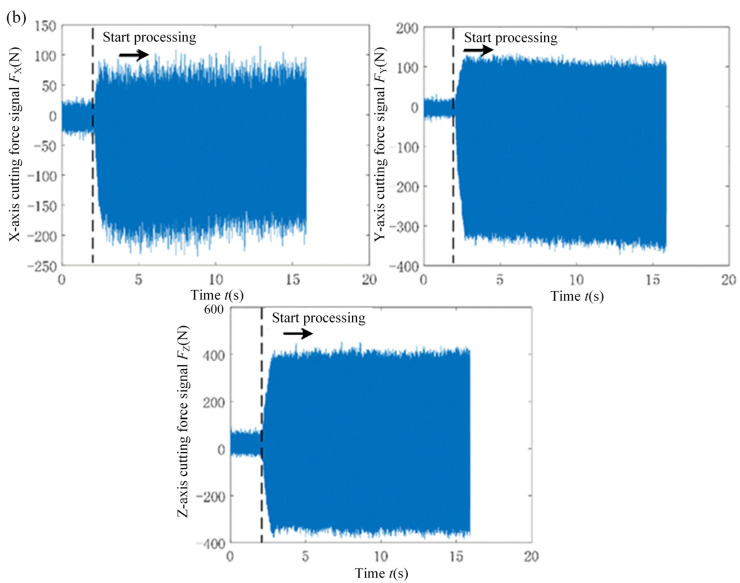
(**a**) Conventional flank milling; (**b**) Rotary ultrasonic flank milling.

Firstly, the cutting force signal in flank milling is analyzed. Figure 6 displays the real-time cutting force signals obtained from traditional flank milling and rotary ultrasonic flank milling. The cutting force is measured under conventional flank milling and rotary ultrasonic flank milling at a feed of 0.01 mm/z and a cutting depth of 0.05 mm. The sound pressure amplitude in rotary ultrasonic flank milling is set at 51 Pa. The cutting force in the *X* direction represents the feed direction of flank milling, the *Y* direction corresponds to the cutting direction of flank milling, and the *Z* direction aligns with the axial direction of the tool. Observing the figure, it’s noticeable that within the traditional flank milling process, the disparity between the maximum and minimum values of the cutting force signal envelope gradually increases from a relatively small value to a stable alteration, taking approximately 10 s to transition. Within this 10 s duration, the progressive variation in cutting force highlights the instability in the contact between the cutting edge and the workpiece. Nevertheless, the imposition of ultrasonic vibration notably abbreviates the transition time from initial machining to stable machining. This indicates that the addition of ultrasonic vibration enhances processing stability, which, in turn, promotes the reduction of surface roughness. The direction of ultrasonic vibration application is a linear reciprocating movement along the *Z*-axis. In actual machining, there are no significant separation characteristics on the *X* and *Y* axes, causing the peak variation of cutting forces to be insignificant. However, there is additional friction between the tool and workpiece along the *Z*-axis, resulting in an increase in cutting forces in the *Z*-axis direction.

**Figure 7:**
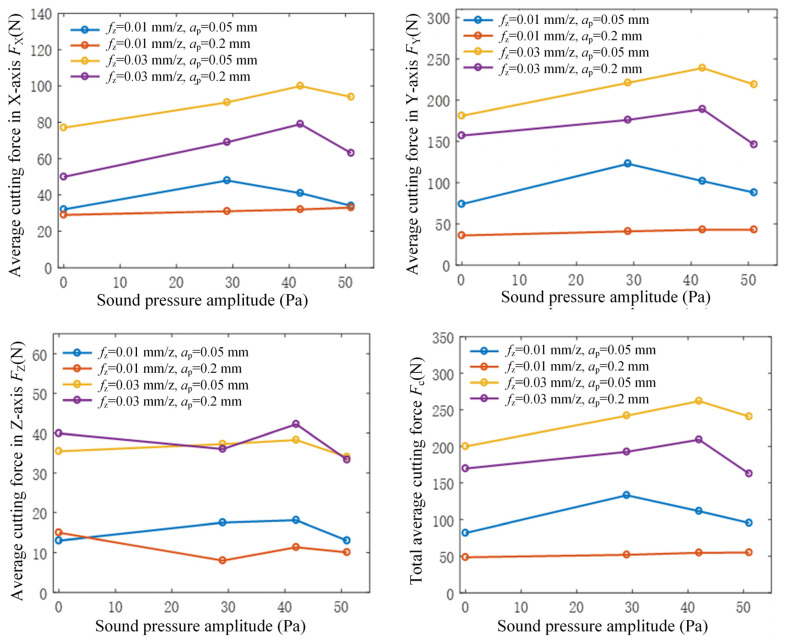
Relationship between cutting force and amplitude of sound pressure radiated from the tool.

Figure 7 illustrates the curves depicting the average cutting force and total cutting force in three directions as the ultrasonic vibration increases under various milling parameters in flank milling, calculated from the cutting force signal averages throughout the machining process. The total average cutting force *F_c_* in the figure is calculated using $F_{c}=\sqrt{{F_{X}}^{2}+{F_{Y}}^{2}+{F_{Z}}^{2}}$. In flank milling, the material is primarily removed by the side edge of the tool, resulting in minimal contact between the tool’s bottom edge and the workpiece. Consequently, the average cutting force in the axial direction is the smallest, followed by the average cutting force in the feed direction, while the largest average cutting force occurs in the cutting direction. In rotary ultrasonic milling, ultrasonic vibration elicits varying effects on cutting forces in different directions. The alteration in ultrasonic vibration primarily influences the average cutting forces in the feed and cutting directions, while displaying minimal impact on the average cutting force in the axial direction. It can be observed from the figure that the superposition of ultrasonic vibration will increase the average cutting force in machining under small amplitudes. With the increase in ultrasonic amplitude, the average cutting force will decrease. This phenomenon highlights that, in flank milling, augmenting the amplitude of ultrasonic vibration effectively diminishes cutting forces during machining, enhances machining stability, and yields high-quality surfaces, thereby maximizing the advantages offered by the rotary ultrasonic machining method.

**Figure 8:**
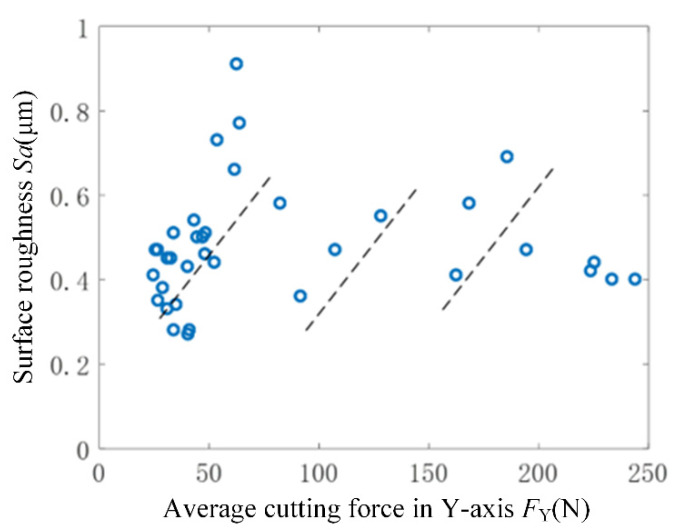
Relationship between surface roughness *Sa* and average cutting force. The blue circles are the original data points of the surface roughness and cutting force relationship, and the dashed line is the line segment of the fitted data points.

Figure 8 depicts the correlation between the average cutting force in the cutting direction during machining and the surface roughness of the workpiece surface. Observing Figure 8 reveals that the workpiece’s surface roughness corresponds to a smaller cutting force during the machining process, resulting in a smaller *Sa* value. Upon local analysis, it becomes evident that the surface roughness increases with the rise in average cutting force, as indicated by the dotted line in the figure. However, within a broader range, the surface roughness exhibits fluctuations corresponding to changes in the average cutting force. There is no discernible positive correlation between surface roughness and the average cutting force. In machining, a smaller cutting force aids in minimizing workpiece deformation, enhancing machining stability, and consequently improving the workpiece’s surface roughness. However, larger cutting forces don’t inherently escalate surface roughness. The magnitude of the cutting force alone doesn’t entirely dictate the workpiece’s surface roughness.

**Figure 9:**
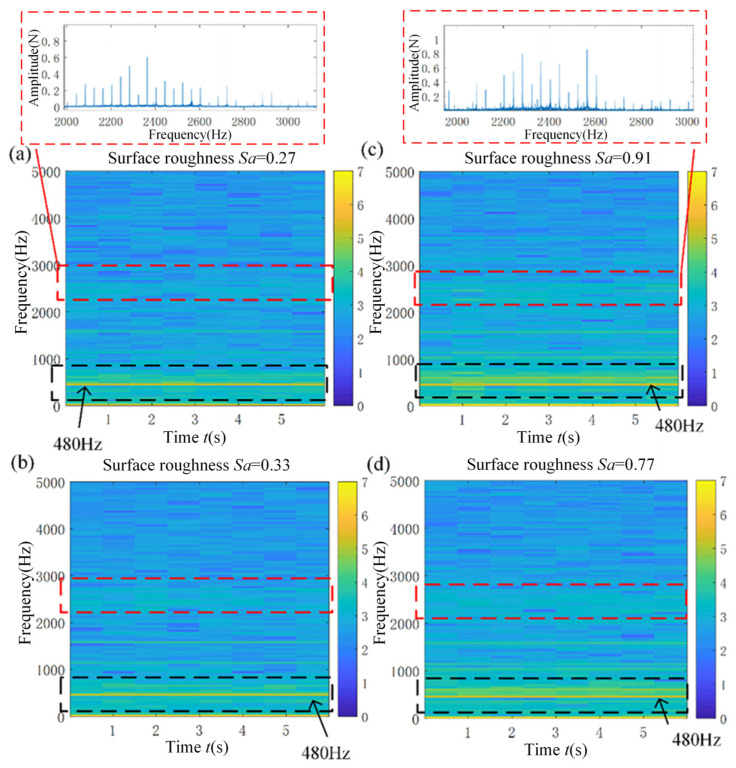
Time-frequency diagram of the cutting force signal in the machining process corresponding to workpieces with different surface roughness. (**a**) Surface roughness *Sa* = 0.27; (**b**) Surface roughness *Sa* = 0.33; (**c**) Surface roughness *Sa* = 0.91; (**d**) Surface roughness *Sa* = 0.77.

In addition to focusing on the cutting force magnitude, delving into the frequency domain distribution of the cutting force signal reveals the intricate interaction between the tool and the workpiece. This utilization of frequency domain analysis provides an alternative perspective, facilitating a deeper exploration of the relationship between cutting force and surface roughness. To comprehensively explore the time-frequency domain characteristics of the cutting force signals and their correlation with different surface roughness conditions, we employed the time-frequency domain analysis method. This method scrutinizes the cutting force signals across different surface roughness levels, resulting in the time-frequency diagram shown in Figure 9. This diagram is derived from the short-time Fourier transform analysis of the cutting force signal during the machining process.

Figure 9 illustrates notable variations in the distribution of cutting force signals within the frequency domain corresponding to different surface roughness levels. During machining, notable components include the 4-fold component of the cutter rotation frequency and a frequency band ranging from 2 kHz to 3 kHz, for instance. In Figure 9a, the cutting force component at 480 Hz measures 46 N, displaying relatively concentrated energy at the adjacent frequencies. In Figure 9c, the cutting force component at 480 Hz registers at 70 N, displaying a relatively divergent energy in the adjacent frequencies. This component exceeds the cutting force signal in the 2 kHz to 3 kHz band shown in Figure 9a. The presence of this frequency component is likely attributed to the fluctuations in cutting force induced by tool-workpiece material friction. Furthermore, the high-frequency component of the cutting force in Figure 9c,d, corresponding to surfaces with higher roughness, appears relatively larger than the time-frequency domain information displayed in Figure 9a,b, corresponding to surfaces with lower roughness.

Based on Figure 8 and Figure 9, it can be concluded that the surface roughness of the workpiece processed by rotary ultrasonic flank milling is closely related to the magnitude and frequency domain characteristics of the cutting force signal. Therefore, when analyzing the mechanism of surface roughness formation in rotary ultrasonic milling, it is necessary to simultaneously analyze the average cutting force and dynamic cutting force characteristics. In addition, it is difficult to have real-time detection of the surface morphology of the workpiece during the process of rotary ultrasonic machining. By monitoring the cutting force, the surface roughness of the workpiece can be indirectly monitored. The time-frequency domain diagram of cutting force signals can serve as an effective visualization tool for online monitoring of changes in the surface roughness of workpieces.

### 3.3. Effect of Tool Vibration on Workpiece Surface Microhardness

**Figure 10:**
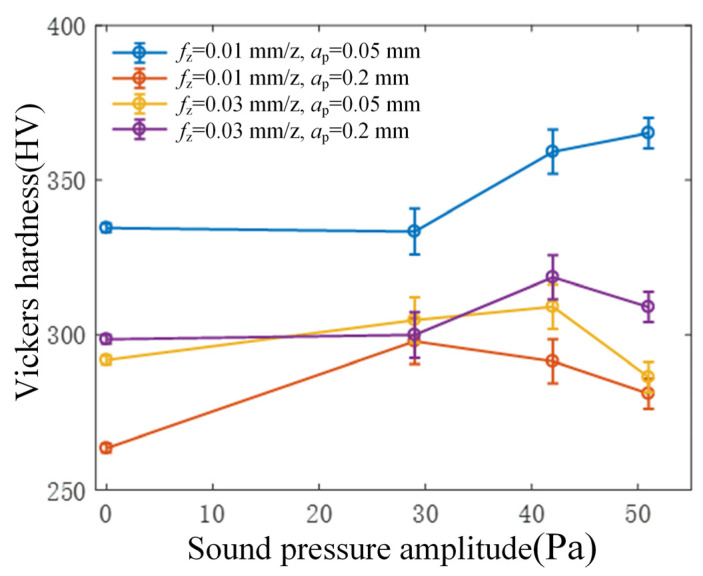
Relationship between the amplitude of sound pressure radiated from the tool and the microhardness.

The graph depicted in Figure 10 illustrates the correlation between the magnitude of ultrasonic vibration and the resulting surface microhardness of the workpiece during flank milling. Employing rotary ultrasonic machining in flank milling demonstrates a capacity to augment the surface microhardness of the machined area. Overall, a discernible pattern emerges: as the intensity of ultrasonic vibration amplifies, a corresponding increase in the surface microhardness of the workpiece becomes evident. However, upon reaching a specific critical threshold in ultrasonic vibration, the microhardness either stabilizes or indicates a marginal decline.

Moreover, a discernible trend emerges from the figure, indicating that the surface microhardness of the workpiece is notably influenced by the feed per tooth, while the impact of cutting depth appears relatively less pronounced. As per the principles of metal cutting [8], the surface microhardness generally increases with an augmented feed rate. As the feed rate increases, the strain rate of the material during the cutting process increases, making the strain hardening effect of the material more pronounced, thereby leading to an increase in the material’s microhardness. In this experimental setup, the surface microhardness of the workpiece achieved under the cutting conditions of 0.01 mm/z feed per tooth and 0.05 mm cutting depth notably surpasses the microhardness obtained under other cutting parameter configurations. Comparable findings were also noted in the research conducted by Zhang [20] during the end milling of nickel-based alloys. This similarity in outcomes might be attributed to the dominance of extrusion deformation as the primary mode of material removal under smaller feed rates and cutting depths. Consequently, this process augments the plastic hardening of the workpiece surface, thereby contributing to the heightened microhardness within the surface layer.

### 3.4. Influence of Tool Vibration on Residual Stress of Workpiece Surface

**Figure 11:**
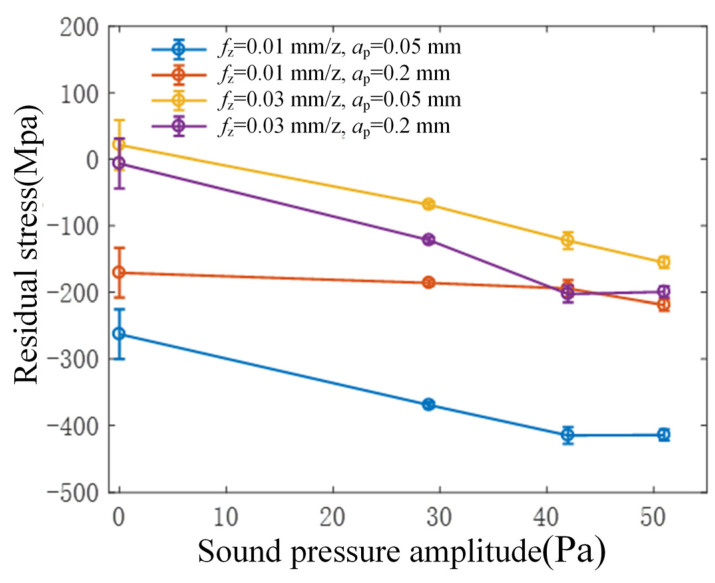
Relationship between the amplitude of sound pressure radiated from the tool and the residual stress.

Figure 11 illustrates the correlation between the amplitude of ultrasonic vibration and the residual stress in the workpiece’s surface layer during flank milling. The obtained results indicate the presence of compressive residual stress on the workpiece’s surface in rotary ultrasonic flank milling, showing a gradual increase in residual compressive stress with the augmentation of ultrasonic vibration. As ultrasonic vibration increases, there is a gradual rise in residual compressive stress on the workpiece’s surface. By combining these findings with the earlier experimental results indicating an increase in surface residual stresses during face milling, it can be deduced that the high-frequency ironing and rubbing between the cutting edge and the workpiece material contribute to heightened residual compressive stresses in rotary ultrasonic milling. This phenomenon holds true whether the ultrasonic vibration of the tool is predominantly along the direction of the workpiece surface or along the direction normal to the workpiece surface. Consequently, this enhancement in residual compressive stresses serves to improve the fatigue strength of the workpiece to a certain extent.

## 4. Conclusions

This paper delves into investigating the influence and mechanism of tool vibration on surface topography and the mechanical properties of the surface layer in rotary ultrasonic flank milling of 40Cr steel. The findings offer valuable insights for the practical application of RUM in engineering. The primary conclusions drawn are as follows:

(1) Compared to traditional flank milling, rotary ultrasonic flank milling can effectively reduce the surface roughness of the workpiece, with a reduction of approximately 40%. Furthermore, as the amplitude of ultrasonic vibration increases, the surface roughness value of the workpiece surface shows a decreasing trend.
(2) From the perspective of cutting force analysis, when ultrasonic vibration is applied, the transition time from the initial machining to a stable cutting force significantly shortens. This indicates that the addition of ultrasonic vibration enhances cutting stability, thus favoring a reduction in surface roughness. In comparison to traditional cutting conditions, the addition of ultrasonic vibration at a low amplitude increases the average cutting force. However, as the amplitude of ultrasonic vibration increases, the average cutting force decreases. Therefore, it can be inferred that increasing the amplitude of ultrasonic vibration during machining effectively reduces cutting forces, enhances machining stability, and achieves high-quality surfaces.
(3) Through analyzing the relationship between cutting force signals and workpiece surface roughness in rotary ultrasonic flank milling, it was observed that there is a close connection between the surface roughness of the workpiece and both the average cutting force and frequency domain characteristics. This suggests utilizing time-frequency domain graphs as a visual tool for real-time monitoring of changes in workpiece surface roughness.
(4) Rotary ultrasonic flank milling can enhance the microhardness of the machined surface. With increased ultrasonic vibration, there is a tendency for an increase in the workpiece’s surface microhardness. When the ultrasonic vibration surpasses a certain critical value, the change in microhardness becomes insignificant or shows a decreasing trend. Additionally, as the ultrasonic vibration amplifies, the residual compressive stress on the surface gradually increases.

## Figures and Tables

**Table 1:** Tool Related Parameters.

Tool Type	Total Length	Helix Angle	Diameter	Blade Length
Four-edge end mill	75 mm	35°	12 mm	30 mm

**Table 2:** Process Parameters of the Flank Milling Experiment.

Experiment Serial Number	Feed per Tooth (*f*_z_) mm/z	Radial Depth of Cut (*a*_e_) mm	Sound Pressure Amplitude (*P*) Pa	Cutting Speed (*V*) mm/min	Axial Depth of Cut (*a*_p_) mm
1–4	0.01	0.05	0, 29, 42, 51	90.5	10
5–8	0.01	0.1	0, 29, 42, 51	90.5	10
9–12	0.01	0.2	0, 29, 42, 51	90.5	10
13–16	0.02	0.05	0, 29, 42, 51	90.5	10
17–20	0.02	0.1	0, 29, 42, 51	90.5	10
21–24	0.02	0.2	0, 29, 42, 51	90.5	10
25–28	0.03	0.05	0, 29, 42, 51	90.5	10
29–32	0.03	0.1	0, 29, 42, 51	90.5	10
33–36	0.03	0.2	0, 29, 42, 51	90.5	10

**Table 3:** Workpiece surface topography of traditional flank milling and rotary ultrasonic flank milling.

	*P* = 0 Pa	*P* = 29 Pa	*P* = 42 Pa	*P* = 51 Pa
No. 1 *f*_z_ = 0.01 mm/z *a*_e_ = 0.05 mm	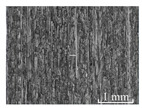	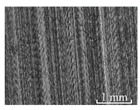	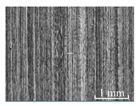	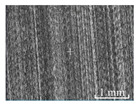
No. 2 *f*_z_ = 0.01 mm/z *a*_e_ = 0.1 mm	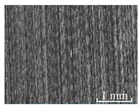	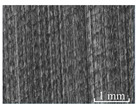	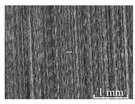	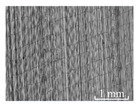
No. 3 *f*_z_ = 0.01 mm/z *a*_e_ =0.2 mm	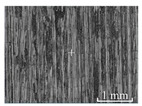	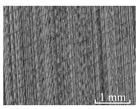	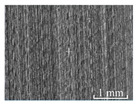	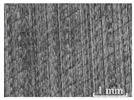
No. 4 *f*_z_ = 0.02 mm/z *a*_e_ = 0.05 mm	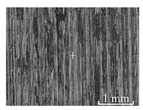	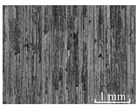	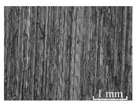	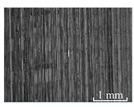
No. 5 *f*_z_ = 0.02 mm/z *a*_e_ = 0.1 mm	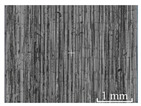	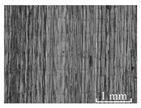	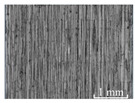	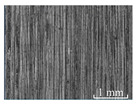
No. 6 *f*_z_ = 0.02 mm/z *a*_e_ = 0.2 mm	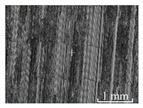	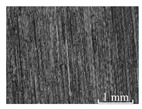	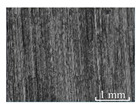	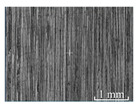
No. 7 *f*_z_ = 0.03 mm/z *a*_e_ = 0.05 mm	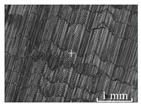	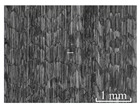	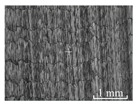	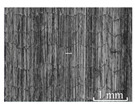
No. 8 *f*_z_ = 0.03 mm/z *a*_e_ = 0.1 mm	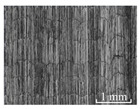	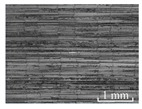	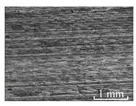	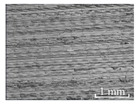
No. 9 *f*_z_ = 0.03 mm/z *a*_e_ = 0.2 mm	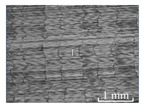	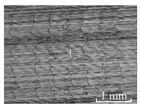	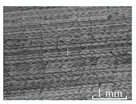	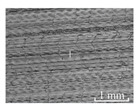

## Data Availability

Data are contained within the article.
